# Microencapsulation of cholic acid via complex coacervation enhances sustained release and antibacterial activity against *Escherichia coli*

**DOI:** 10.3389/fvets.2026.1874445

**Published:** 2026-06-10

**Authors:** Xiuling He, Ganggang Qu, Danqi Shen, Jing Lu, Haobo Li, Wei Mao, Zhipeng Jia

**Affiliations:** 1 Inner Mongolia Key Laboratory of Veterinary Fundamentals and Disease Prevention and Control for Herbivorous Livestock, Hohhot, China; 2 Key Laboratory of Clinical Diagnosis and Treatment Techniques for Animal Disease, Ministry of Agriculture, Hohhot, China

**Keywords:** antibacterial activity, cholic acid, complex coacervation, *Escherichia coli*, microencapsulation

## Abstract

This study aimed to develop a microencapsulated cholic acid (CA) preparation to improve the stability, sustained-release performance, and antibacterial activity of CA for potential use in antibiotic-free animal production. CA microcapsules were prepared by complex coacervation using gelatin and gum arabic as wall materials, and the formulation was optimized by single-factor and orthogonal experimental designs. The optimal preparation conditions were a wall material concentration of 2.5%, a core-to-wall ratio of 1.8:1, and pH 4.1, yielding a drug loading rate of 49.73% and an encapsulation efficiency of 88.76%. The microcapsules showed spherical morphology, low hygroscopicity, improved thermal and storage stability, and a sustained-release profile over 72 h in simulated gastrointestinal media. Both free CA and microencapsulated CA significantly inhibited *Escherichia coli* growth (*p* < 0.001), while the microcapsules provided a more prolonged antibacterial effect consistent with controlled release. These findings indicate that complex coacervation is a practical strategy for improving CA performance and support the potential use of CA microcapsules as antimicrobial feed additives in livestock production.

## 1 Introduction

The widespread use of antibiotics in livestock production has contributed significantly to the emergence of antimicrobial resistance and the accumulation of drug residues in animal-derived products, posing serious risks to animal health, food safety, and public health. Consequently, the development of effective and sustainable alternatives to antibiotics has become a critical priority in veterinary science and animal husbandry.

Cholic acid (CA), a primary bile acid synthesized in the liver, is widely present in the bile of mammals such as cattle, sheep, and pigs (1, 2). Chemically, CA is defined as 3α, 7α, 12α-trihydroxy-5β-cholanic acid (C_24_H_40_O_5_), and it typically exists as a free bile acid with a steroidal structure. The pharmacological effects of CA span multiple levels. In addition to its well-known role in emulsifying dietary lipids and facilitating fat digestion, CA has been reported to exhibit antibacterial, anti-inflammatory, and immunomodulatory activities (3). Recent studies have further highlighted its involvement in regulating bile acid metabolism, Lipid and glucose homeostasis, and immune responses, indicating its potential as a functional additive in animal production systems (28). Despite these promising biological functions, the practical application of CA is limited by its poor water solubility, low stability, and susceptibility to environmental factors, which reduce its bioavailability and effectiveness (4).

Microencapsulation has emerged as an effective strategy to overcome these limitations by improving the stability, bioavailability, and controlled-release properties of bioactive compounds. This technology involves the encapsulation of active substances (core materials) within protective wall materials to form micro-scale particles, thereby minimizing exposure to adverse environmental conditions such as oxygen, light, and moisture (5–8). Furthermore, microencapsulation enables sustained and targeted release of encapsulated compounds, enhancing their functional performance and reducing the frequency of administration. The selection of appropriate wall materials is crucial, as it directly influences encapsulation efficiency, release kinetics, and overall stability. Based on the source and characteristics, wall materials can mainly be classified into three categories: natural polymer wall materials, synthetic polymer wall materials, and composite wall materials. For example,gum arabic,chitosam and polylactic acid, each possessing distinct physicochemical properties (9).

Among the various microencapsulation techniques, complex coacervation is a widely applied physicochemical method that relies on electrostatic interactions between oppositely charged polymers (10, 11). The complex copolymerization process is usually spontaneous or induced by altering physicochemical parameters such as pH, temperature (12). This technique typically involves the formation of a coacervate phase through charge neutralization, followed by solidification to produce stable microcapsules with low water solubility but excellent controlled-release performance (13). Gelatin (GE), a positively charged protein derived from collagen, and gum arabic (GA), a negatively charged natural polysaccharide, are commonly used as wall materials in this process due to their excellent biocompatibility, emulsifying properties, and stability (14–16). Their complementary charge characteristics facilitate efficient encapsulation and contribute to desirable release profiles.

Although CA has demonstrated promising biological activities, studies on its application as a functional feed additive in livestock production remain limited. In particular, there is a lack of research focusing on improving its physicochemical properties and evaluating its antibacterial performance through advanced delivery systems. Therefore, the present study aimed to develop CA microcapsules using a gelatin–gum arabic complex coacervation method, optimize the preparation conditions, and systematically characterize their physicochemical properties, stability, and *in vitro* release behavior. In addition, the antibacterial activity of the microencapsulated CA against *Escherichia coli* was evaluated to assess its potential as an alternative to antibiotics in livestock production.

## 2 Materials and methods

### 2.1 Materials

Cholic acid (CA) was extracted at the Laboratory of Veterinary Pharmacology and Toxicology, Inner Mongolia Agricultural University, and stored in sealed containers at room temperature until use. Gelatin-gum arabic, employed as wall materials for microencapsulation, were obtained from Shanghai Yuanye Biotechnology Co., Ltd. and Shanghai Aladdin Biochemical Technology Co., Ltd., respectively. All other chemicals and reagents were of analytical grade and purchased from Sinopharm Chemical Reagents Co., Ltd., Shanghai Yuanye Biotechnology Co., Ltd., and Tianjin Kemao Chemical Reagents Co., Ltd. All materials were used without further purification unless otherwise specified.

### 2.2 Preparation of Cholic acid microcapsules by complex Coacervation

CA microcapsules were prepared using the complex coacervation method, with gelatin and gum arabic as wall materials. Briefly, gelatin was hydrated in distilled water and dissolved at 50 °C in a water bath. Concurrently, gum arabic was dispersed in water and stirred until fully dissolved. CA (100 mg) was dissolved in anhydrous ethanol and gradually mixed with aliquots of gelatin and gum arabic solutions under continuous stirring for 1 h to remove visible crystals. The remaining gelatin and gum arabic solutions were then incorporated and homogenized. The mixture was maintained at 50 °C while 5% acetic acid solution was added dropwise to adjust the pH to the target value. Microcapsule formation was monitored microscopically. After dilution with water at 30 °C, the suspension was cooled in an ice-water bath to below 10 °C, followed by crosslinking with 0.375 mL of 25% glutaraldehyde solution for 2 h. The resulting microcapsules were stored at −80 °C and freeze-dried to yield the final product.

### 2.3 Single factor experiment

To identify the optimal conditions for microcapsule preparation, single-factor experiments were conducted to systematically evaluate the effects of core-to-wall ratio (1.8, 1, 2.7, 1, 3.6,1), capsule material concentration (gelatin and gum arabic solutions at 2.5–2.5%, 3.0–3.0%, and 3.5–3.5%), and coagulation pH (4.0, 4.1, 4.2) on drug loading. All experiments were performed in triplicate, and the mean drug loading rate, reported to two decimal places, was used for data analysis.

Specifically, the effect of core-to-wall ratio on the encapsulation efficiency of CA microcapsules was assessed at a 3.0% gelatin–gum arabic (G–A) composite concentration, coagulation pH 4.0, reaction temperature 30 °C, and reaction time of 60 min. The influence of capsule material concentration was examined at a fixed core-to-wall ratio of 1.8:1 under identical pH, temperature, and reaction time conditions. In addition, the effect of coagulation pH on microcapsule formation was evaluated at a 3.0% G–A composite concentration, core-to-wall ratio of 1.8:1, reaction temperature of 30 °C, and reaction time of 60 min.

The results of these single-factor experiments provided a robust basis for the subsequent orthogonal experimental design, enabling the identification of the conditions that maximize encapsulation efficiency and drug loading.

### 2.4 Orthogonal experiment

Based on the outcomes of the single-factor experiments, an orthogonal experimental design was employed to further optimize the preparation conditions of cholic acid microcapsules. A three-factor, three-level orthogonal array was implemented, with capsule material concentration (Factor A), core-to-wall ratio (Factor B), and coagulation pH (Factor C) as the independent variables. The encapsulation efficiency of the microcapsules served as the primary evaluation index. The specific factor levels and combinations used in the orthogonal design are summarized in Table 1.

**Table 1:** Design of the orthogonal experiment.

Level	Factors		
	A: capsule material concentration	B: core-to-wall ratio	C: coagulation pH
1	2.5% (A1)	1.8:1 (B1)	4.0 (C1)
2	3.0% (A2)	2.7:1 (B2)	4.1 (C2)
3	3.5% (A3)	3.6:1 (B3)	4.2 (C3)

### 2.5 Determination of physicochemical properties of the powders

#### 2.5.1 Moisture content and Hygroscopicity

Moisture content was measured according to AOAC (American Association of Analytical Chemists) standard procedures. Hygroscopicity was determined by exposing 0.1 g of freeze-dried microcapsules in a desiccator containing saturated sodium sulfate solution (RH 81%) for 7 days, and expressed as the percentage of water absorbed per 100 g of sample.

#### 2.5.2 Fourier transform infrared spectroscopy (FTIR) and X-ray diffraction (XRD)

FTIR spectra of gelatin, gum arabic, CA, and CA microcapsules were recorded to confirm encapsulation. XRD analysis was performed to assess crystallinity and structural changes post-encapsulation.

#### 2.5.3 Drug loading rate and encapsulation efficiency

For solution preparation, a reference stock solution was prepared by dissolving cholic acid (CA) in 60% acetic acid to a final concentration of 4.28 mg/mL. The test solution was prepared by transferring 1 mL of settled microcapsule supernatant into a 15 mL centrifuge tube, adding 14 mL of 50% sulfuric acid, and heating the mixture at 75 °C with shaking for 18 min, followed by cooling to room temperature. The reference stock solution was treated under identical conditions, and 200 μL aliquots of each solution were transferred into a 96-well microplate for absorbance measurement. A blank control, consisting of 1 mL of 60% acetic acid, was processed in parallel.

Encapsulation efficiency (EE) and drug loading (DL) were calculated as follows. A standard curve was constructed using the reference solution, and the CA content in the test solution was determined spectrophotometrically. EE was calculated using the formula:

EE (%) = Amount of drug encapsulated in microcapsules/Total amount of drug (encapsulated + unencapsulated) × 100.

For determination of drug loading, the microcapsule supernatant was discarded after settling, and the remaining microcapsules were stored at −80 °C and freeze-dried. A 0.02 g sample of freeze-dried microcapsules was suspended in 100 μL distilled water, mixed with 400 μL ethanol, and sonicated at 70 W for 10 min. Subsequently, 4.2 mL of sulfuric acid was added, and the solution was processed as described above for absorbance measurement. DL was calculated according to:

DL (%) = Drug content in microcapsules/Total weight of microcapsules×100.

All measurements were performed in triplicate to ensure accuracy and reproducibility.

#### 2.5.4 Surface morphology and particle size

Microcapsule morphology was examined using scanning electron microscopy (SEM), and particle size distribution was analyzed using a laser particle size analyzer under wet conditions.

#### 2.5.5 Thermal and storage stability

Differential scanning calorimetry (DSC) was performed to analyze the thermal properties of gelatin, gum arabic, cholic acid, and the prepared microcapsules, including initial temperature, peak temperature, and melting point (T_m_), under a nitrogen atmosphere. Samples of 10–20 mg were accurately weighed, sealed in aluminum pans, cooled with liquid nitrogen, and heated from 10 °Cto 200 °Cat a rate of 10 °C/min.

For storage stability evaluation, microcapsules were vacuum-sealed and stored under two conditions: 7 °C in a refrigerator and 37 °C in an incubator. Freeze-dried samples from the same container were collected weekly over a period of 7 weeks to determine total CA content. Measures were taken to minimize exposure to light and oxygen during storage, ensuring accurate assessment of microcapsule stability and drug retention.

#### 2.5.6 In vitro release studies

Microcapsule release profiles were evaluated using dialysis bags diffusion method. Briefly, dialysis bags with a molecular weight cutoff (MWCO) of 8,000 were used to contain the microcapsules, which were immersed in simulated gastric fluid (pH 1.3) or intestinal fluid (pH 6.8) at 37 °C with magnetic stirring. Samples were withdrawn at predetermined intervals, replaced with fresh medium, and analyzed spectrophotometrically to determine CA release.

#### 2.5.7 High-temperature stability

Microcapsules were stored at 60 °C for 10 days, and changes in appearance and drug loading were recorded on days 5 and 10 to assess thermal resilience.

### 2.6 Statistical analysis

All data were expressed as mean±standard deviation (SD). One-way ANOVA followed by Tukeyˈs post-hoc test was performed using SAS 9.1.3 to evaluate statistical significance, with *p* < 0.05 considered significant.

## 3 Results

### 3.1 Optimization of Cholic acid microcapsules

**Figure 1:**
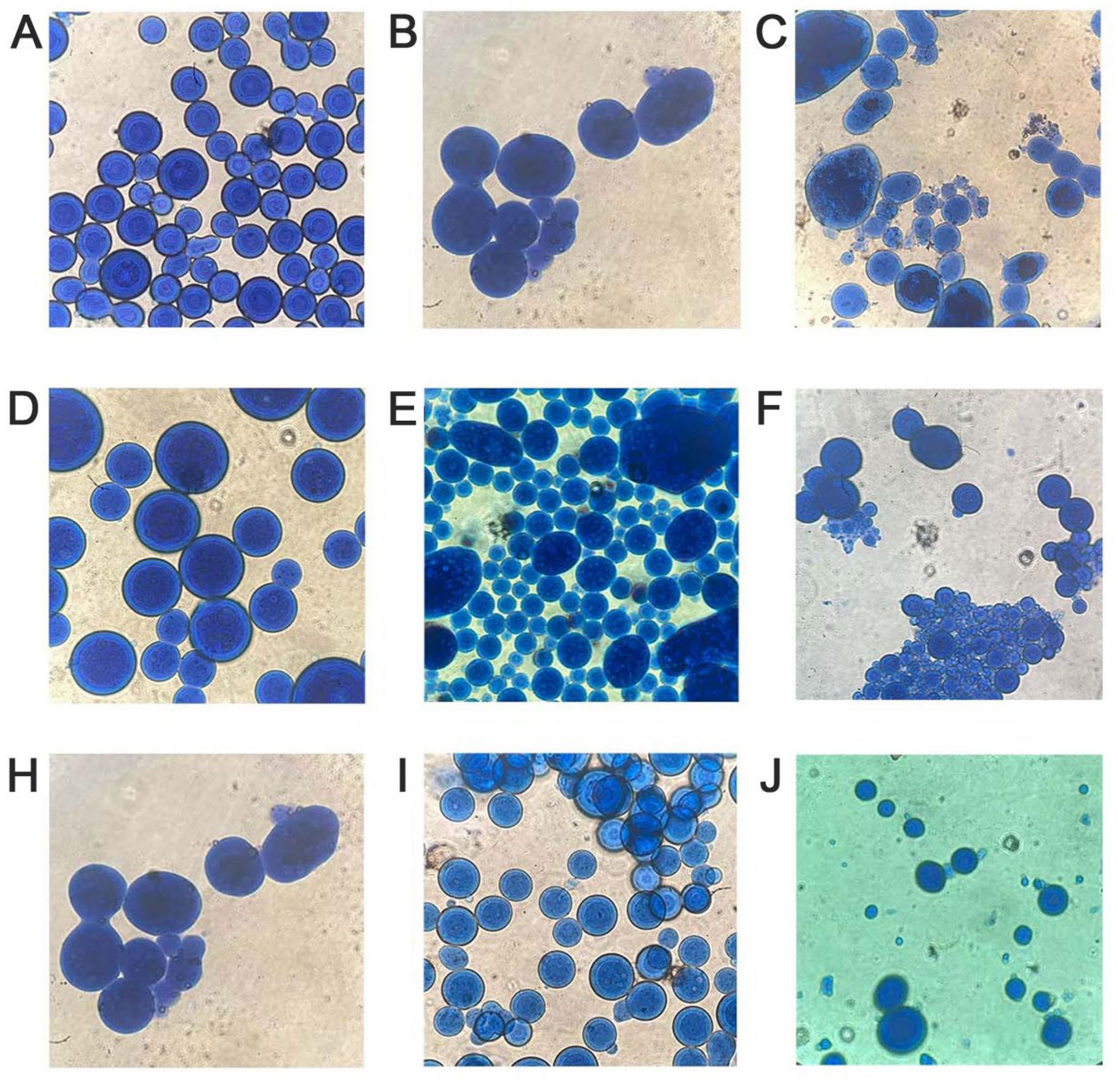
Optical microscopy images of cholic acid microcapsules at various core-to-wall ratios, capsule material concentrations, and pH values (400 × magnification). **(A)** Core-to-wall ratio of 1.8:1; **(B)** Core-to-wall ratio of 2.7:1; **(C)** Core-to-wall ratio of 3.6:1; **(D)** Capsule material concentration of 2.5%; **(E)** Capsule material concentration of 3.0%; **(F)** Capsule material concentration of 3.5%; **(H)** pH 4.0; **(I)** pH 4.1; **(J)** pH 4.2.

The preparation conditions of cholic acid microcapsules were systematically optimized through single-factor and orthogonal experiments to maximize drug loading efficiency. Single-factor experiments evaluated the effects of core-to-wall ratio, capsule material concentration, and coagulation pH (Table 2, Figures 1A–J).

**Table 2:** Single-factor effects on Cholic acid microcapsules.

Factor	Level	Drug loading rate (%)
Core-to-wall ratio	1.8:1	48.73 ± 0.52
	2.7:1	45.83 ± 0.79
	3.6:1	47.21 ± 0.21
Capsule material concentration (%)	2.5	48.73 ± 0.17
	3.0	44.43 ± 1.31
	3.5	49.12 ± 0.42
Coagulation pH	4.0	45.43 ± 0.37
	4.1	45.99 ± 0.45
	4.2	42.12 ± 1.21

For the core-to-wall ratio, a 1.8:1 ratio achieved the highest drug loading (48.73 ± 0.52%) with uniform microcapsules, while 2.7:1 (45.83 ± 0.79%) and 3.6:1 (47.21 ± 0.21%) produced lower loading and unevenly distributed microcapsules (Figures 1A–C). Regarding capsule material concentration, 2.5% gelatin–gum Arabic (G–A) yielded slightly lower drug loading (48.73 ± 0.17%) but resulted in uniform spherical microcapsules without aggregation, whereas 3.0 and 3.5% increased loading to 44.43 ± 1.31% and 49.12 ± 0.42% respectively, though aggregation was observed (Figures 1D–F). Coagulation pH significantly influenced microcapsule formation, with pH 4.1 producing the highest drug loading (45.99 ± 0.45%), compared to 45.43 ± 0.37% at pH 4.0 and 42.12 ± 1.21% at pH 4.2 (Figures 1H–J). Based on these results, factor levels for the orthogonal design were set at: core-to-wall ratio 1.8:1, capsule material concentration 2.5%, and pH 4.0–4.2.

The orthogonal experimental design further assessed the combined effects of these three factors across nine experimental groups (Table 3). Analysis of range (Rj) and mean values (kj) revealed that the factors influencing drug loading followed the order: pH > core-to-wall ratio > capsule material concentration, with Rj values of 0.0891, 0.0812, and 0.0105, respectively, indicating pH as the most critical factor. Variance analysis (Table 4) confirmed that all three factors significantly affected drug loading (*p* < 0.05).

**Table 3:** Orthogonal experiment results for cholic acid microcapsules.

Run	Capsule material concentration	Capsule core ratio	pH	Drug loading rate (%)
1	2.5%	1.8:1	4.0	48.73 ± 0.27
2	2.5%	2.7:1	4.1	55.39 ± 0.31
3	2.5%	3.6:1	4.2	29.19 ± 1.2
4	3.0%	1.8:1	4.1	45.99 ± 0.45
5	3.0%	2.7:1	4.2	41.20 ± 1.70
6	3.0%	3.6:1	4.0	45.45 ± 1.21
7	3.5%	1.8:1	4.2	49.50 ± 0.72
8	3.5%	2.7:1	4.0	35.45 ± 0.42
9	3.5%	3.6:1	4.1	45.22 ± 0.15
Kj1	1.3330	1.4422	1.2963	
Kj2	1.3264	1.3204	1.4660	
Kj3	1.3016	1.1986	1.1989	
kj1	0.4444	0.4807	0.4321	
kj2	0.4421	0.4401	0.4887	
kj3	0.4339	0.3995	0.3996	
Rj	0.0105	0.0812	0.0891	

**Table 4:** Variance analysis of orthogonal experiment.

Factor	SS	df	F	*p*-value	Significance
A	53.67	2	26.835	*p*<0.05	*
B	3.405	1	2.191	*p*<0.05	*
C	4.382	2	3.405	*p*<0.05	*
Error	0	1	—	—	—

*indicates significant difference (*p* < 0.05); SS, sum of squares; df, degrees of freedom.

The optimal combination identified was A1B1C2—capsule material concentration 2.5%, core-to-wall ratio 1.8:1, and pH 4.1. Validation through three parallel batches produced drug loading rates of 48.73, 49.87, and 49.71%, with a mean of 49.44%, confirming the robustness and reproducibility of the optimized parameters.

### 3.2 Physicochemical and structural characterization of Cholic acid microcapsules

**Figure 2:**
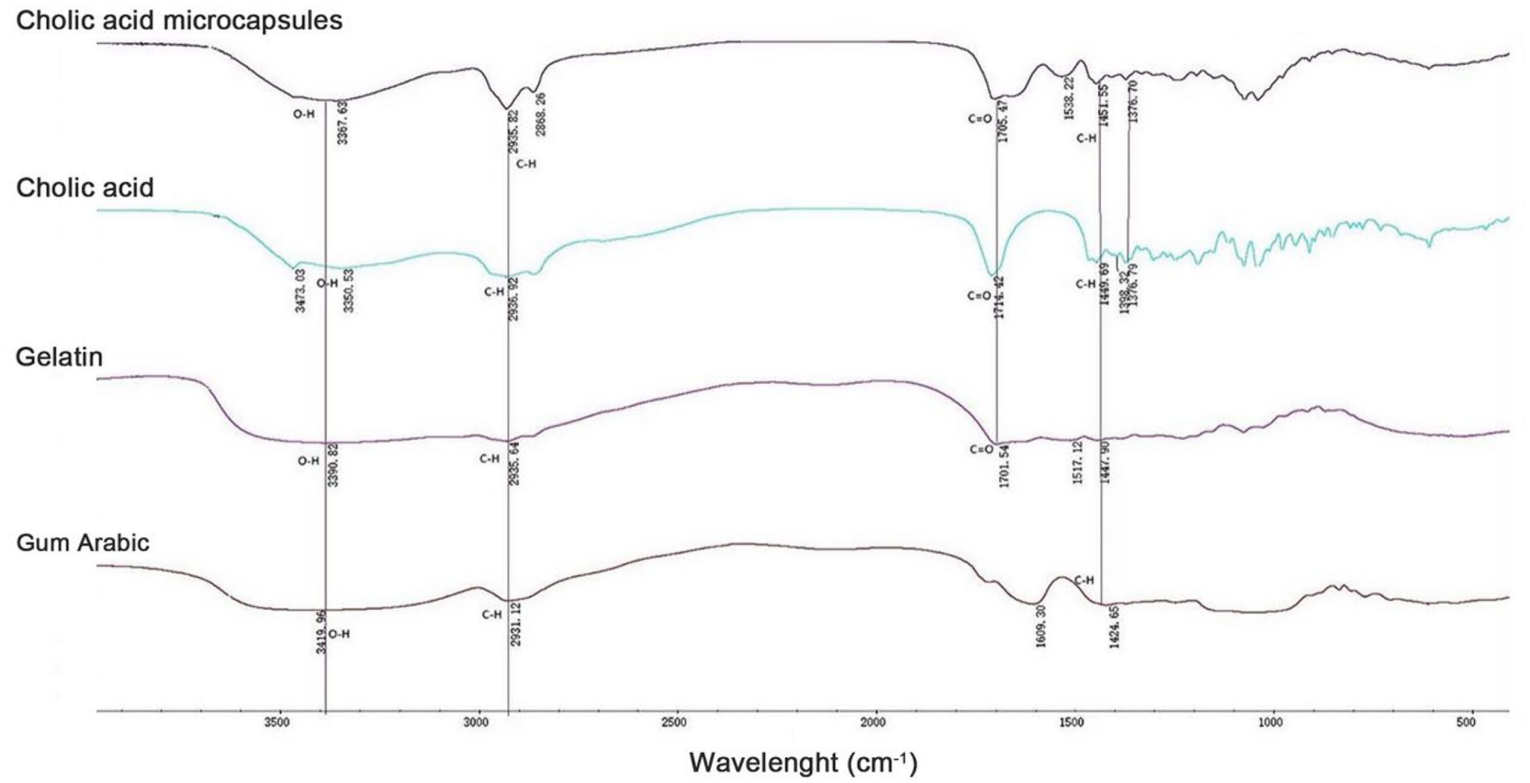
Fourier transform infrared (FTIR) spectra of cholic acid microcapsules fabricated via complex coacervation method.

**Figure 3:**
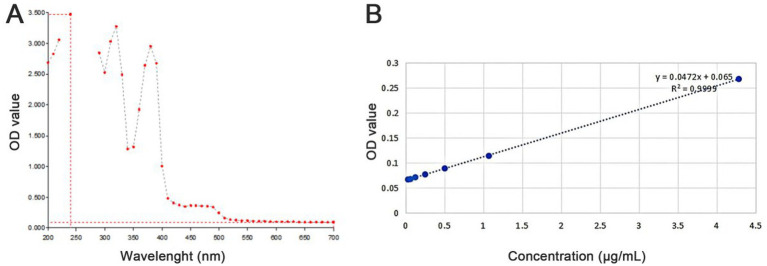
Spectroscopic analysis of cholic acid. **(A)** Determination of the absorption wavelength of cholic acid; **(B)** Quantification of cholic acid content.

**Figure 4:**
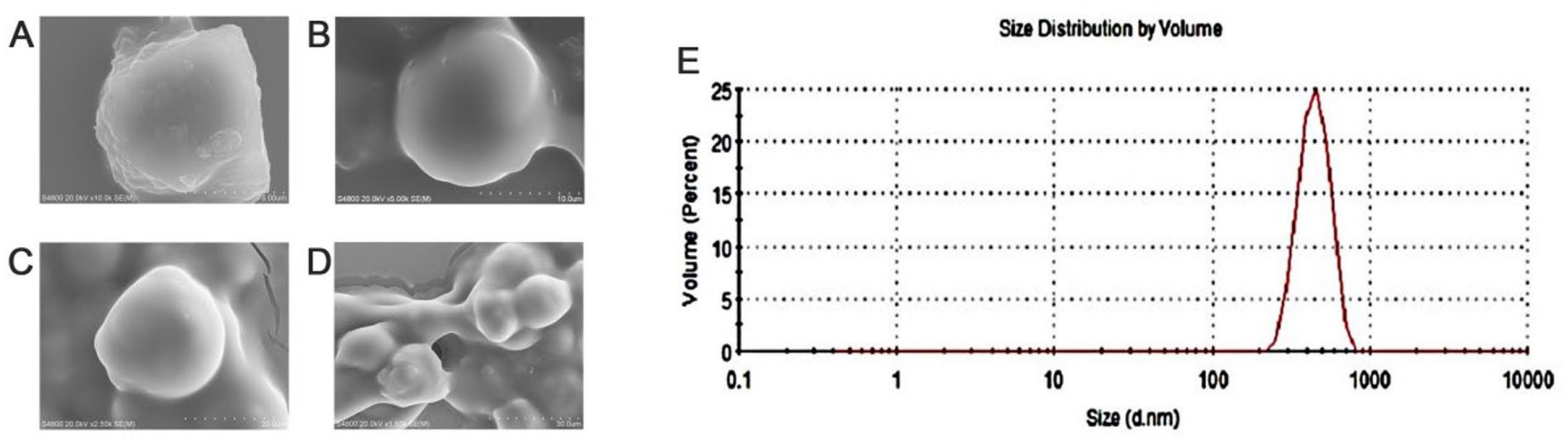
Morphological and particle size analysis of cholic acid microcapsules. **(A–D)** Scanning electron microscopy (SEM) image of cholic acid microcapsules fabricated via the complex coacervation method; **(E)** Particle size distribution of cholic acid microcapsules prepared by the complex coacervation method.

**Figure 5:**
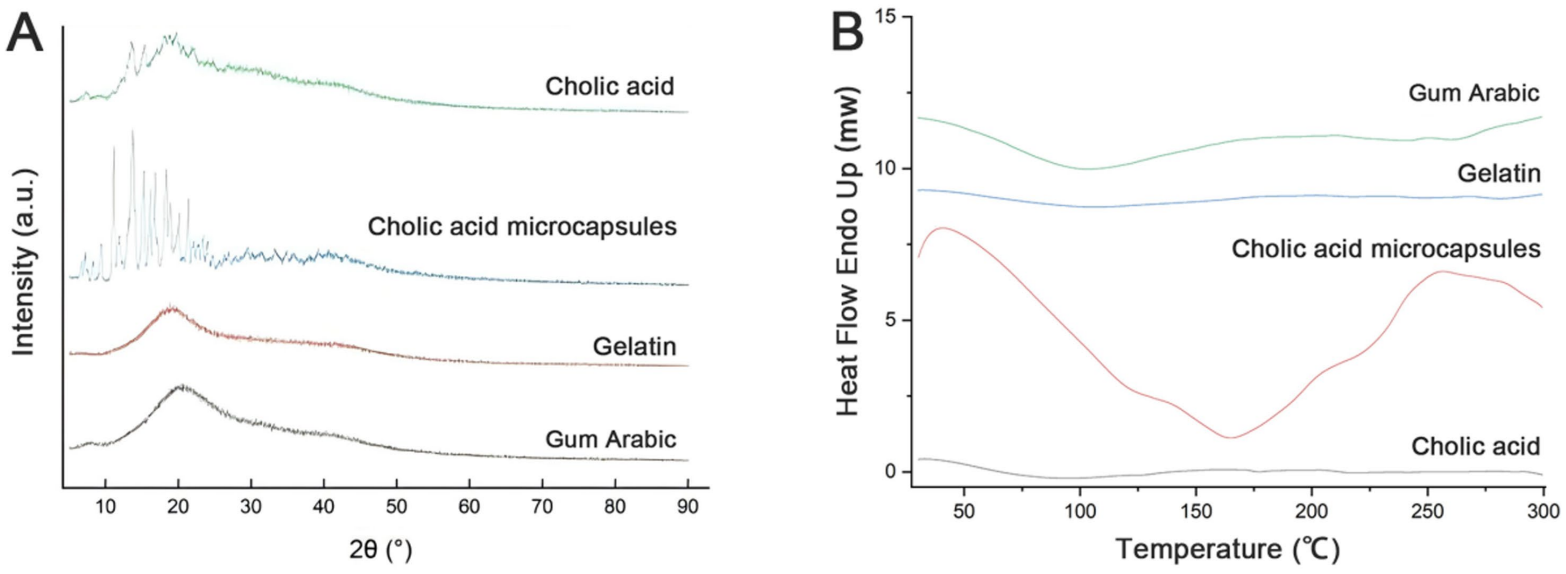
**(A)** Structural and thermal characterization of cholic acid microcapsules. X-ray diffraction (XRD) spectra; **(B)** Differential scanning calorimetry (DSC) thermogram.

The physicochemical properties and structural characteristics of CA microcapsules, prepared using the optimized formulation from single-factor and orthogonal experiments, were systematically evaluated (Table 5, Figures 2–5). The optimized microcapsules exhibited a drug loading rate of 49.73 ± 0.72% and an encapsulation efficiency of 88.76 ± 0.45%, exceeding the >80% threshold stipulated by the Chinese Pharmacopoeia (2020 Edition). Moisture content was negligible (0 ± 0%), and hygroscopicity was low (7 ± 0.0012%), reflecting the stability of the freeze-dried microcapsules and their minimal tendency for agglomeration under standard storage conditions.

**Table 5:** Physicochemical properties of cholic acid microcapsules.

Parameter	Value (%)
Moisture content	0 ± 0
Moisture absorption	7 ± 0.0012
Drug loading rate	49.73 ± 0.72
Encapsulation efficiency	88.76 ± 0.45

Data are expressed as mean ± SD (*n* = 3).

Fourier Transform Infrared (FTIR) spectroscopy confirmed the successful encapsulation of cholic acid within the microcapsule matrix (Figure 2). Characteristic absorption peaks of gelatin (N–H at 3390.82 cm^−1^, C=O at 1701.54 cm^−1^, N–H bending of amide II at 1517.12 cm^−1^) and gum arabic (O–H at 3419.96 cm^−1^, alkyl–CH_2_ at 2831.12 cm^−1^, carboxyl C=O at 1609.30 cm^−1^) were preserved, while microcapsules displayed corresponding O–H stretching at 3367.63 cm^−1^, C–H stretching at 2935.82 cm^−1^, and C=O stretching at 1705.47 cm^−1^, consistent with the presence of all components and indicating effective encapsulation of CA.

CA content was quantified spectrophotometrically at 380 nm, the wavelength identified as optimal based on spectral analysis (Figure 3A). The standard curve (y = 0.0472x + 0.065, *R^2^* = 0.9999) demonstrated excellent linearity over 0.032–4.28 mg/mL, ensuring precise and reliable quantification (Figure 3B).

Surface morphology examined via scanning electron microscopy (SEM, Figure 4A–D) revealed approximately spherical microcapsules with smooth surfaces and minimal porosity. Some particle adhesion was observed, consistent with gelatin–gum arabic wall material properties. Most microcapsules were below 10 μm in diameter and exhibited uniform, fully spherical morphology, indicating effective encapsulation and structural integrity. Particle size distribution analysis (Figure 4 E) demonstrated a range of 200.05–800.10 nm, with a mean particle size of 451.3 nm, highlighting the efficacy of the complex coacervation method in producing small, uniform microcapsules.

X-ray diffraction (XRD) patterns (Figure 5A) revealed broad, diffuse peaks for gelatin, gum arabic, cholic acid, and microcapsules, confirming predominantly amorphous structures. The freeze-drying process preserved this amorphous state, preventing crystallization and enhancing solubility and hygroscopicity, while indicating that storage conditions must be carefully controlled to maintain long-term physical stability.

### 3.3 Thermal and storage stability of cholic acid microcapsules

Differential scanning calorimetry (DSC) analysis revealed a peak temperature (Tp) of 169.57 °C for free cholic acid, whereas the Tp values for microcapsules, gelatin, and gum arabic exceeded 300 °C (Figure 5B). This marked increase in Tp demonstrates that encapsulation substantially enhances the thermal stability of cholic acid by protecting the core material from heat-induced degradation.

**Figure 6:**
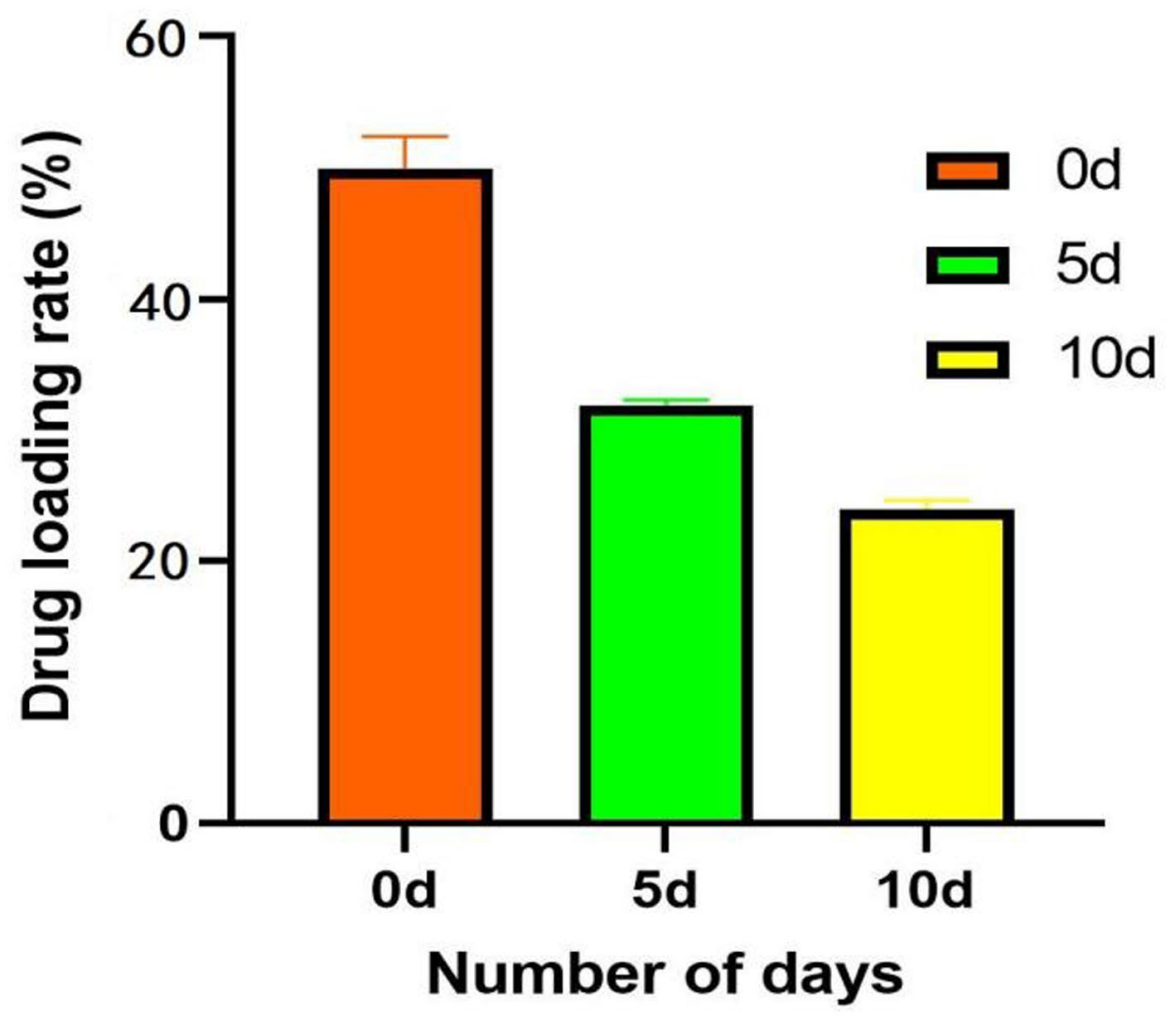
High-temperature stability profile of cholic acid microcapsules.

The high-temperature stability of microcapsules was further evaluated at 60 °C over 10 days (Figure 6). No significant changes in appearance or drug loading were observed during the first 5 days. However, by day 10, slight yellowing and minor agglomeration were detected, accompanied by a gradual decrease in drug loading. These results indicate that although encapsulation improves resistance to thermal and oxidative degradation, prolonged exposure to elevated temperatures still negatively affects microcapsule stability.

**Figure 7:**
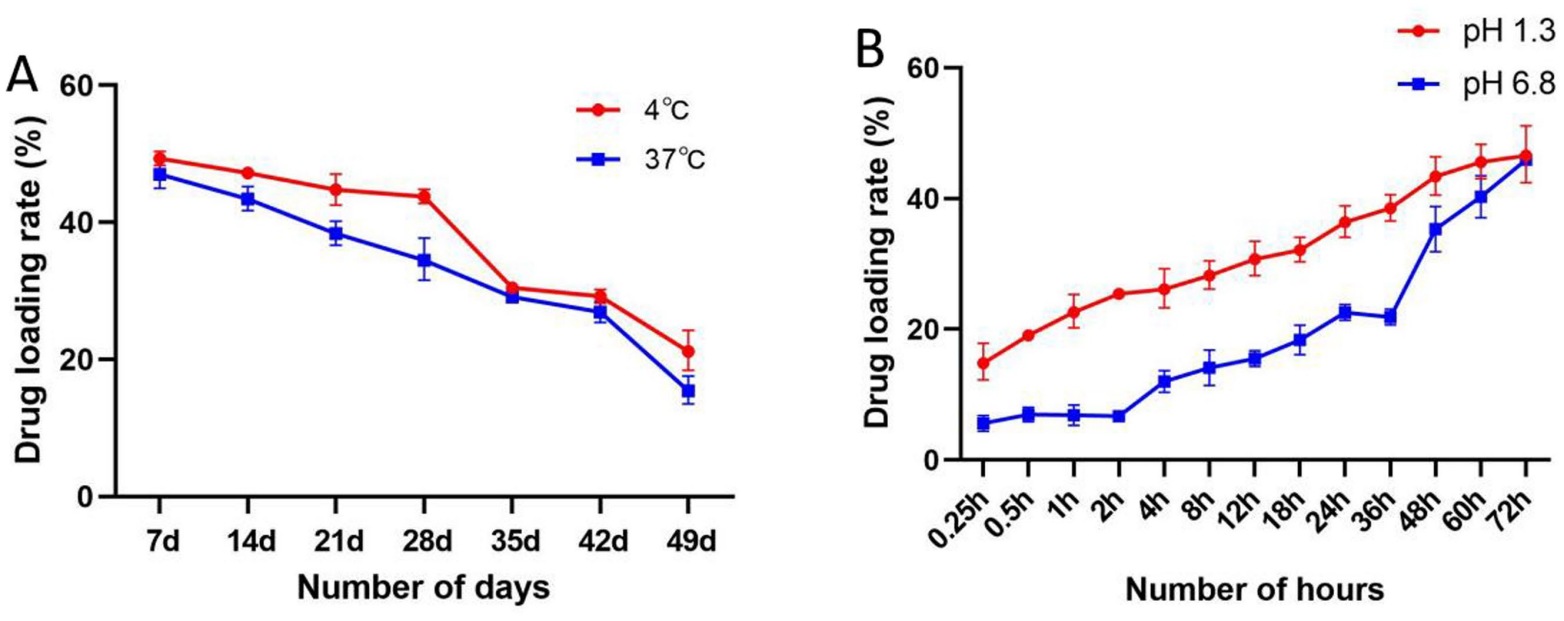
Stability and release profiles of cholic acid microcapsules. **(A)** Stability curves of cholic acid microcapsules fabricated via the complex coacervation method; **(B)** Release curves of cholic acid microcapsules fabricated via the complex coacervation method.

Storage stability was further assessed at 4 °C and 37 °C over an extended period (Figure 7A). The drug loading of microcapsules decreased progressively with increasing storage time under both conditions. Notably, microcapsules stored at 4 °C consistently retained higher drug loading compared to those stored at 37 °C at corresponding time points, indicating superior stability under low-temperature conditions.

These findings demonstrate that microencapsulation significantly enhances the thermal stability of cholic acid; however, both elevated temperature and prolonged storage can lead to gradual degradation. Therefore, it is recommended to use low-temperature storage, placing the microcapsules in a dry, cool and dark environment to maintain its integrity and protect the biological activity of CA.

### 3.4 *In vitro* release evaluation results of Cholic acid microcapsules

The release rate data of cholic acid microcapsules, prepared by the complex coacervation method, in dissolution media at pH 1.3 and pH 6.8 are presented in Tables 6. The release curve of these microcapsules is shown in Figure 7B. From Figure 7B, it is evident that the cholic acid microcapsules required 72.0 h to release 45.90% of the cholic acid loading capacity at pH 1.3, while at pH 6.8, the encapsulated cholic acid was almost completely released by 72.0 h. This result is highly consistent with the physiological pH gradient of the gastrointestinal tract: In the stomach (acidic), drug release is inhibited, reducing irritation to the gastric mucosa, and at the same time preventing bile acids from prematurely degrading in an acidic environment; In the intestine (neutral), the drug is released continuously and stably, prolonging the duration of action and enhancing bioavailability.

**Table 6:** In vitro cumulative release of cholic acid microcapsules in simulated gastric (pH 1.3) and intestinal (pH 6.8) fluids over 72 h.

Time (h)	Cumulative release (%)	
	pH1.3	pH6.8
0.25	14.83 ± 1.73	5.65 ± 1.00
0.5	19.07 ± 0.00	7.77 ± 1.00
1	24.72 ± 5.99	7.77 ± 2.00
2	25.42 ± 0.00	7.06 ± 1.00
4	26.13 ± 1.00	12.01 ± 2.00
8	26.13 ± 5.56	14.12 ± 3.60
12	31.07 ± 1.00	15.54 ± 1.00
18	29.66 ± 2.64	18.36 ± 2.64
24	31.78 ± 6.08	22.60 ± 1.00
36	37.43 ± 4.99	21.89 ± 1.00
48	41.67 ± 3.00	35.31 ± 3.60
60	45.20 ± 1.00	40.25 ± 3.46
72	45.90 ± 11.98	48.02 ± 3.99

### 3.5 Antibacterial activity of cholic acid and microcapsules

**Figure 8:**
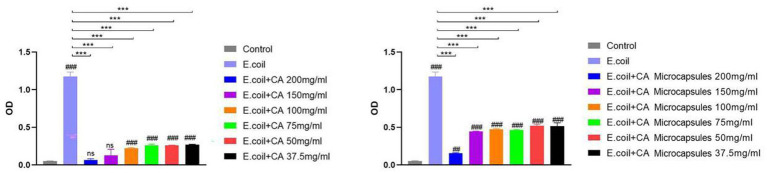
*In vitro* antibacterial experiment of cholic acid and cholic acid microcapsules. ***p < 0.001, ###*p* < 0.001. #represents comparisons with control group and * represents comparison between groups: No significant difference.

The *Escherichia coli* (ATCC 25922) used in the experiment was purchased from the Institute of Microbiology, Chinese Academy of Sciences. The concentration of the bacterial culture was 1.0 × 10^6^ CFU/mL. The antibacterial effects of free cholic acid and cholic acid microcapsules against *Escherichia coli* were evaluated at multiple concentrations (Figure 8). Free cholic acid significantly inhibited bacterial growth compared with the control (*p* < 0.001). Microcapsules also exhibited significant inhibition (*p* < 0.001 vs. control), though slightly less than free cholic acid at equivalent concentrations, likely due to incomplete release within the first 18–24 h. Moreover, the carrier materials of microcapsules (such as polysaccharide or polymer shells) may partially block direct contact between CA and bacteria. This delayed release results in a lower initial effective concentration, reducing the efficiency of CA in directly disrupting bacterial cell membranes. Dose-dependent antibacterial effects were observed, confirming that microencapsulation maintains functional activity while providing controlled release.

## 4 Discussion

Cholic acid, a steroidal organic acid predominantly present in its free form, plays well-established physiological roles, including emulsification of dietary fats and modulation of antipyretic and anti-inflammatory pathways (17). Previous studies have also demonstrated that cholic acid exhibit significant advantages in antibacterial and antiviral aspects (18). Despite these documented properties, the potential of cholic acid to mitigate bacterial diarrhea in young ruminants remains underexplored (1, 2). Accordingly, developing microencapsulated CA holds promise for controlling bacterial infections in aquaculture, thereby contributing to sustainable and environmentally responsible production.

In this study, cholic acid content was quantified via ultraviolet–visible (UV–Vis) spectrophotometry at 380 nm, consistent with prior findings (26). Method validation demonstrated a robust linear relationship, confirming the specificity and reliability of the assay. Microcapsules were prepared using complex coacervation, yielding particles with superior morphological and functional properties (19). Single-factor experiments systematically examined the effects of core-to-wall ratio, capsule material concentration, and pH on drug loading, providing essential data for orthogonal optimization (20, 21). The optimal preparation parameters were identified as 2.5% gelatin–gum arabic, a core-to-wall ratio of 1.8:1, and pH 4.1, consistent with prior studies (22).

Physicochemical characterization revealed key insights into microcapsule performance (23). Freeze-dried microcapsules exhibited negligible moisture content (0%) and low hygroscopicity (7%), reflecting effective prevention of agglomeration, which could otherwise impede release. Drug loading (49.73%) and encapsulation efficiency (88.76%) exceeded previously reported values (24), likely due to differences in macromolecular composition and core-to-wall ratios. These outcomes were influenced by multiple factors, including wall material selection, physicochemical properties of cholic acid, temperature, and pH.

Morphological analysis using scanning electron microscopy (SEM) confirmed predominantly spherical microcapsules, in contrast to the irregular structures reported by Das, where surfactants or organic solvents may have affected particle uniformity. X-ray diffraction (XRD) revealed amorphous structures across all components and microcapsules, corroborating observations that the amorphous state enhances stability at both ambient and elevated temperatures (25). Particle size distribution was narrow and normally distributed, indicating high uniformity.

Differential scanning calorimetry (DSC) demonstrated reduced heat flux for microcapsules relative to free cholic acid, indicative of improved thermal resilience. These findings contrast with reports of higher thermal permeability in other methods (25), highlighting the suitability of the present approach for thermally labile compounds. Storage studies showed greater drug retention at 4 °Ccompared to 37 °C, consistent with the susceptibility of cholic acid to thermal degradation and confirming the protective effect of encapsulation (25). Elevated temperatures induce polymer phase transitions—crystalline to amorphous, glassy to rubbery, solid to molten, or sol to gel—which can alter release dynamics (26).

*In vitro* release studies revealed a sustained-release profile, significantly attenuated compared to free cholic acid, allowing prolonged delivery in simulated gastric (pH 1.3) and intestinal (pH 6.8) environments. This sustained release reduces administration frequency, enhancing safety and practicality. High-temperature storage tests indicated that elevated conditions compromise microcapsule stability and antioxidant activity, consistent with observations of thermal degradation in encapsulated compounds (27).

Overall, the study successfully encapsulated cholic acid in gelatin and gum arabic via complex concertation, optimizing conditions through single-factor and orthogonal experiments. Comprehensive evaluations—including physicochemical properties, morphology, FTIR, XRD, DSC, storage stability, *in vitro* release, and antibacterial activity—confirmed the efficacy and stability of the microcapsules. These results provide a robust theoretical foundation for the application of cholic acid microcapsules as functional feed additives, with significant potential to improve aquaculture health management and reduce reliance on conventional antibiotics. Furthermore, the sample size and research scale of the *in vivo* experiments should be expanded to conduct a more in-depth analysis of their safety performance and the regulatory response of the intestinal flora, while also verifying the actual feeding application effects.

## 5 Conclusion

This study demonstrates that cholic acid can be effectively microencapsulated using gelatin and gum arabic via complex coacervation, producing microcapsules with high drug loading (49.73 ± 0.72%) and encapsulation efficiency (88.76 ± 0.45%). Single-factor and orthogonal experiments identified optimal preparation conditions, resulting in microcapsules with uniform spherical morphology, narrow particle size distribution (mean 451.3 nm), and predominantly amorphous structures. The microcapsules exhibited excellent thermal stability, sustained-release properties under simulated gastrointestinal conditions, and significant antibacterial activity against *Escherichia coli*, while storage at low temperatures maintained drug integrity over time.

Compared with conventional Nano emulsions, the microcapsule formulation developed in this study effectively overcomes the drawbacks of weak sustained-release performance, high costs and poor thermodynamic stability. Microencapsulation achieves controlled-release performance and functional antibacterial efficacy, providing a robust foundation for these application as feed additives in livestock and aquaculture. These findings highlight the potential of microencapsulation technology to enhance the bioavailability and efficacy of bioactive compounds, offering an effective alternative to conventional antibiotics for improving animal health.

## Data Availability

The original contributions presented in the study are included in the article/supplementary material, further inquiries can be directed to the corresponding author.
